# Visualizing energy transfer between redox-active colloids

**DOI:** 10.1126/sciadv.ady7716

**Published:** 2025-09-03

**Authors:** Alan Subing Qu, Zihao Ou, Yavuz Savsatli, Lehan Yao, Yu Cao, Hao Yu, Elena C. Montoto, Jingshu Hui, Bo Li, Julio A. N. T. Soares, Lydia Kisley, Brian P. Bailey, Elizabeth A. Murphy, Junsheng Liu, Jennifer Huang, Christopher M. Evans, Charles M. Schroeder, Joaquín Rodríguez-López, Jeffrey S. Moore, Qian Chen, Paul V. Braun

**Affiliations:** ^1^ Department of Materials Science and Engineering, University of Illinois Urbana-Champaign, Urbana, IL 61801, USA.; ^2^ Materials Research Laboratory, University of Illinois Urbana-Champaign, Urbana, IL 61801, USA.; ^3^ Beckman Institute for Advanced Science and Technology, University of Illinois Urbana-Champaign, Urbana, IL 61801, USA.; ^4^ Grainger College of Engineering, University of Illinois Urbana-Champaign, Urbana, IL 61801, USA.; ^5^ Department of Physics, University of Texas at Dallas, Richardson, TX 75080, USA.; ^6^ Department of Chemistry, University of Illinois Urbana-Champaign, Urbana, IL 61801, USA.; ^7^ Department of Chemical and Biomolecular Engineering, University of Illinois Urbana-Champaign, Urbana, IL 61801, USA.; ^8^ Department of Mechanical Engineering, Kennesaw State University, Kennesaw, GA 30114, USA.

## Abstract

Redox-active colloids (RACs) represent a novel class of energy carriers that exchange electrical energy upon contact. Understanding contact-mediated electron transfer dynamics in RACs offers insights into physical contact events in colloidal suspensions and enables quantification of electrical energy transport in nonconjugated polymers. Redox-based electron transport was directly observed in monolayers of micron-sized RACs containing ethyl-viologen side groups via fluorescence microscopy through an unexpected nonlinear electrofluorochromism that is quantitatively coupled to the redox state of the colloid. Via imaging studies, using this electrofluorochromism, the apparent charge transfer diffusion coefficient *D*_CT_ of the RAC was easily determined. The visualization of energy transport within suspensions of redox-active colloids was also demonstrated. Our work elucidates fundamental mechanisms of energy transport in colloidal systems, informs the development of next-generation redox flow batteries, and may inspire new designs of smart active soft matter including conductive polymers for applications ranging from electrochemical sensors and organic electronics to colloidal robotics.

## Teaser

Visualization of electron transfer between redox-active colloids enables a paradigm shift in studying dynamics of soft matter.

## INTRODUCTION

While electrically conductive conjugated polymers (*1*) have been extensively studied (*2*, *3*), there are fewer studies on nonconjugated electrically conducting polymers (*4*, *5*). Although most nonconjugated polymers are not electrically conductive (*6*, *7*), prior work showed that poly(4-glycidyloxy-2,2,6,6-tetramethylpiperidine-1-oxyl), a radical polymer glass (*8*), exhibited an electrical conductivity of 28 S/m over a distance of 600 nm via a solid-state charge transfer (CT) mechanism. Unlike conjugated polymers, where conduction is exciton-based (*9*) and requires conjugation (*2*) that can be broken by defects, energy transport in nonconjugated systems is defect tolerant. The only requirement is that redox-active pendant groups be in proximity to form a solid-state percolating path or have sufficient mobility to enable electron exchanging contacts (*10*, *11*), as is the case for the ethyl-viologen (EV)–based redox-active colloids (RACs; fig. S1) (*10*), which serve as the basis of this study. We note that, similar to conjugated polymer semiconductors (*12*, *13*), doping (*4*–*6*) can further enhance electrical conductivity of redox-active polymers; to reduce the number of variables, the RACs studied here are not doped.

Most colloid science investigations (*14*–*16*) treat the colloids as inert objects, which exchange at most kinetic energy upon contact. Colloidal systems where particles can also exchange electrical energy have not been deeply studied [although energy exchange is certainly occurring in colloid-based redox flow battery electrolytes (*10*, *17*)]. Polymeric RACs (figs. S1 and S2) are believed to be electrically conductive through exchange of electrons between neighboring redox-active EV groups appended to the polymer backbone. The net electron hopping is primarily driven by the concentration gradient of radical cations (EV^+ •^) and dications (EV^2+^) and exhibits a quasi-diffusional behavior as proposed by Dahms and Ruff (*18*, *19*) (Eq. 1)

$$D=D_{\text{phys}}+D_{\text{CT}}$$ (1)

where the diffusion coefficient, *D*, contains both *D*_phys_ and *D*_CT_ terms. *D*_phys_ represents the physical transport of the polymer backbone (negligible in a crosslinked system such as the RAC studied here), and *D*_CT_ is the CT diffusion coefficient (units of cm^2^/s). *D*_CT_ (cm^2^/s) is a key performance metric indicating how fast electron transport occurs and is typically determined through electrochemical modeling (*10*) or via scanning electrochemical microscopy (*20*) on a single particle. We note that conventional electrical/electrochemical measurements cannot directly determine charge transport distances, therefore measuring interparticle energy transport kinetics requires development of the real-space optical method described here to track the energetic state of assemblies of redox-active systems.

Enabled by the discovery that RACs are electrofluorochromic (*21*, *22*), we directly visualize electrical energy transport both between an underlying electrode and the RAC and within touching monolayers and submonolayers of these RACs. We specifically probe intercolloid energy transport, in contrast to previous reports (*11*, *17*, *20*, *23*), which focus on transport within continuous polymer structures. We determine the effective CT diffusion coefficient (*24*) by observing and quantifying in situ fluorescence patterns moving on the order of 10 μm across a static two-dimensional (2D) colloidal layer only in contact on one side with the electrode. A key advantage of real-space and real-time imaging is the ability to extract full-field data, thereby enabling direct observation on how percolation of the 2D colloidal array relates to energy transfer. We additionally visualize electron transfer upon dynamic colloidal collisions in a suspension of RACs, which may facilitate in-operando mapping of state-of-charge (SOC) of individual particles in redox flow batteries (RFBs) (*25*, *26*). Prior work (*27*, *28*) revealed the importance of direct imaging to understand dynamics occurring within RFBs. For example, in 2022, Kang and coworkers (*27*) used the colorimetric properties of 5,10-bis(2-methoxyethyl)-5,10-dihydrophenazine to visualize the interplay of electrochemistry and hydrodynamics in RFBs.

## RESULTS

### RAC electrofluorochromism

**Fig. 1.**
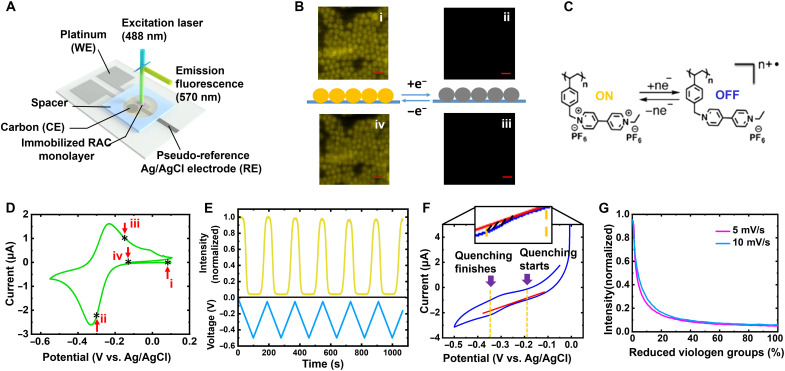
RAC electrofluorochromism. (**A**) Three-electrode characterization cell schematic. (**B**) Fluorescence imaging of RAC monolayer (scale bars, 2 μm) in oxidized (i and iv) and reduced (ii and iii) states. (**C**) Reversible reduction and oxidation of the pendant EV group. The dication EV^2+^ (oxidized) form is fluorescent, and the radical cation EV^+•^ (reduced) form is dark. (**D**) CV on the RAC monolayer over the viologen first electron-transfer voltage range at a sweep rate of 1 mV/s. Fluorescence images presented in (B) are taken at the four indicated points (i, ii, iii, and iv). (**E**) Top: Fluorescence intensity versus time of a field of 166 colloids (fig. S4) at a sweep rate of 5 mV/s. Bottom: Voltage versus time (cycling between −0.05 and −0.5 V versus Ag/AgCl). (**F**) Tangent line drawing (indicatory) for extraction of Faradaic charges going into/out of RACs from the first round of 5 mV/s CV data in fig. S5. Enclosed area (cross-hatched region of zoomed-in image between red tangent line and blue redox peak signatures) is the Faradaic charge. (**G**) First-cycle fluorescence intensity (normalized) versus percent reduced EV groups at sweep rates of 5 and 10 mV/s.

RAC fluorescence is highly dependent on the RAC redox state under 488-nm illumination (fig. S3). Strong fluorescence is observed in the fully oxidized state (EV^2+^), which is largely quenched when only 5 to 10% of the EV within the RAC are reduced to the radical cation state EV^+ •^ (Fig. 1G and movie S1). This visible light electrofluorochromic behavior in a viologen-based system is a notable expansion of the limited number of chemistries shown to be reversibly electrofluorochromic (*29*–*31*).

Using an airtight three-electrode electrochemical cell (Materials and Methods) compatible with fluorescence microscopy (Fig. 1A), cyclic voltammetry (CV) and fluorescence imaging are concurrently performed on an acetonitrile swollen RAC submonolayer at a scan rate of 1 mV/s (Fig. 1B, fig. S6, and movie S1). The RACs were synthesized following our previous publications (*10*, *11*) and were chemically similar to those in our previous reports [compare Fourier transform infrared spectroscopy in figures S2 and S2.3 of (*10*)]. The difference between the RAC reduction and oxidation peaks is less than 100 mV (Fig. 1D and movie S1), suggesting electrochemical reversibility; fluorescence switching is also reversible. Using a ferrocene calibrated pseudo-reference electrode (RE) (Materials and Methods and fig. S7), the redox peaks are found to be associated with cycling between EV^2+^ and EV^+•^ states (Fig. 1C) (*11*). Four fluorescence images in the CV cycle are presented in Fig. 1B. The RACs’ fluorescence disappears during reduction and reappears during oxidation, and fluorescence changes appear to occur across the entire volumes of the colloidal particles.

The relationship between RAC fluorescence emission and SOC is quantitatively determined by casting a few drops of a dilute [~0.04% (w/w)] solution of RACs onto the working electrode (WE) and removing all the colloids outside the boundary of the electrode using an isopropanol-dipped wipe. The number of RACs (fig. S8) participating in Faradaic reactions is precisely counted via a particle-counting method (*32*). Six CV cycles (fig. S5) at 5 mV/s are performed on these 34,731 particles contacting the WE while simultaneously observing the fluorescence of 166 colloids (Fig. 1E). During the reduction sweep, Faradaic charge is extracted by the established tangent-line method (Fig. 1F) (*24*) from a wave superimposed on a sloping baseline of capacitive current (Materials and Methods and fig. S9). Slower scan rates are generally not used because the redox peaks become increasingly buried in the baseline under these conditions (fig. S10), making quantitative analysis unreliable. The initiation of quenching is defined as when the total fluorescence intensity in the field of view decreases by 5%. By combining the Faradaic charge versus time (fig. S11B) and the luminescence intensity versus time (Fig. 1E), fluorescence versus redox state of RACs can be determined (Fig. 1G). Only small differences are observed at 5 and 10 mV/s. Electrochemical data over the entire electrode are integrated to obtain a larger readable current signal while imaging a much smaller number of particles to obtain single-particle fluorescence data. Low-magnification experiments (fig. S12) show that the fluorescence of the majority of colloids (~95%) increases and decreases in unison. Our results suggest that the remaining colloids that do not follow the behavior of the others are in poor contact with the electrode. The working curve for the first cycle is selected because RACs slowly lose their electrochemical reversibility, and the first cycle has a minimum of potentially photobleached particles

$$I_{o}/I=1+K_{s}\left[Q\right]$$ (2)

**Fig. 2.**
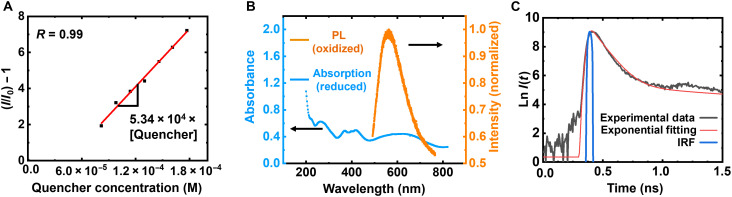
RAC quenching photophysics. (**A**) Stern-Volmer plot of reduced monomer (EV^+•^) quenching oxidized RAC dispersion (10 mM total viologen groups). (**B**) Spectral superimposition of reduced RAC (0.1 mM) absorbance and normalized PL emission for oxidized RAC (under 488-nm excitation). (**C**) Time-resolved PL measurement and single-exponential fit of fluorescence lifetime of oxidized RAC dispersion (0.1 M viologen groups). IRF, instrument response function.

Fluorescence quenching is a strong function of redox state. Fluorescence decreases roughly by ~80% as the first 13% (5 mV/s) to 16% (10 mV/s) of the EV groups are reduced (Fig. 1G), indicating that 1 EV^+ •^ effectively quenches ~4 or 5 EV^2+^ within a nearly fully oxidized RAC. Figure S13 repeats Fig. 1G for cycles 2 and 3 at 5 mV/s. There is a reasonable overlap between subsequent cycles (fig. S13). However, because the Coulombic efficiency is less than 100%, subsequent cycles do not start in the fully oxidized state. Figure 2A shows a Stern-Volmer (*33*) plot obtained by measuring the photoluminescence (PL) intensity at 570 nm of an acetonitrile suspension of oxidized RACs (containing a total of 10 mM EV) as chemically reduced EV monomers (EV^+•^) were added stepwise. We note the PL decreased substantially after only 0.08 mM reduced monomers was added (fig. S14). Assuming quenching follows a Stern-Volmer relationship (Eq. 2), the quenching constant *K*_s_ is obtained through a linear fit (Fig. 2A) and calculated to be 5.34 × 10^4^ M^−1^. Note that the fit did not go through the origin, which we speculate is likely due to an oxidant, probably O_2_, present in the solution that oxidizes some of the added EV^+•^. Another possibility as to why the curve did not go through the origin is positive curvature in the Stern-Volmer plot due to a combination of dynamic and static quenching (*33*). As we did not collect sufficient data at low quencher concentration, we cannot speculate if this is the case. Given that our electrochemical studies are at high effective quencher concentrations, the plotted region of the Stern-Volmer plot is most relevant for the study here. The data in Fig. 2A suggest that one EV^+•^ monomer can quench emission from ~79 EV^2+^ pendant groups on the polymer backbone (Materials and Methods) and that the EV^+•^ monomer diffuses readily into acetonitrile-swollen RACs; otherwise, substantial fluorescence would remain even in the presence of EV^+•^.

We speculate quenching is primarily due to Förster resonance energy transfer (FRET) but cannot completely rule out Dexter energy transfer [the two most common mechanisms for fluorescence quenching (*34*)]. Another possibility is electron transfer; however, we can largely rule that out. FRET requires a spectral overlap between the donor and acceptor species, whereas the Dexter process requires a wave function overlap between them. An important distinction is that FRET operates over distances on the order of 50 Å, while the Dexter process only occurs within distances of ~10 Å. We note that Dexter energy transfer involves a simultaneous exchange of electrons between donor and acceptor molecules and is distinct from the electron transfer discussed here (fig. S15), which is one-directional. As shown in the absorbance data (Fig. 2B), the reduced RAC has an absorbance peak that overlaps the oxidized RAC’s emission peak (centered around 570 nm). Given the high concentration of EV groups in the swollen RAC (order one EV/nm^3^), about 125 EV groups are within a conventional Förster radius of ~50 Å. The spectral overlap between the reduced RAC absorbance peak, the oxidized RAC emission peak, EV concentration, and the Stern-Volmer plot in Fig. 2A, which suggests that 1 EV^+•^ monomer can effectively quench ~79 EV^2+^, makes FRET the most probable quenching mechanism. As prior work (*23*) showed that spacing between neighboring viologen groups is similar to or slightly less than 10 Å, we cannot completely rule out that Dexter energy transfer also plays a role. However, a Dexter mechanism should be limited to quenching of a few oxidized EV groups per reduced EV group. Electron transfer (*21*) is unlikely involved with RAC quenching because time-resolved PL shows a fast fluorescence lifetime of ~0.1 ns (Fig. 2C), along with a much slower decay. A 0.1 ns is many orders of magnitude shorter than the time (*23*) (inversion of the self-exchange rate constant, *k*_EX_, reported for viologen-based redox active polymer, which is ~8.1 × 10^6^ s^−1^) needed to complete even one single-electron self-exchange, indicating the nonlinearity of RAC’s electrofluorochromism is probably due to FRET with perhaps a minor contribution from a Dexter transfer process.

### Interparticle energy transport

**Fig. 3.**
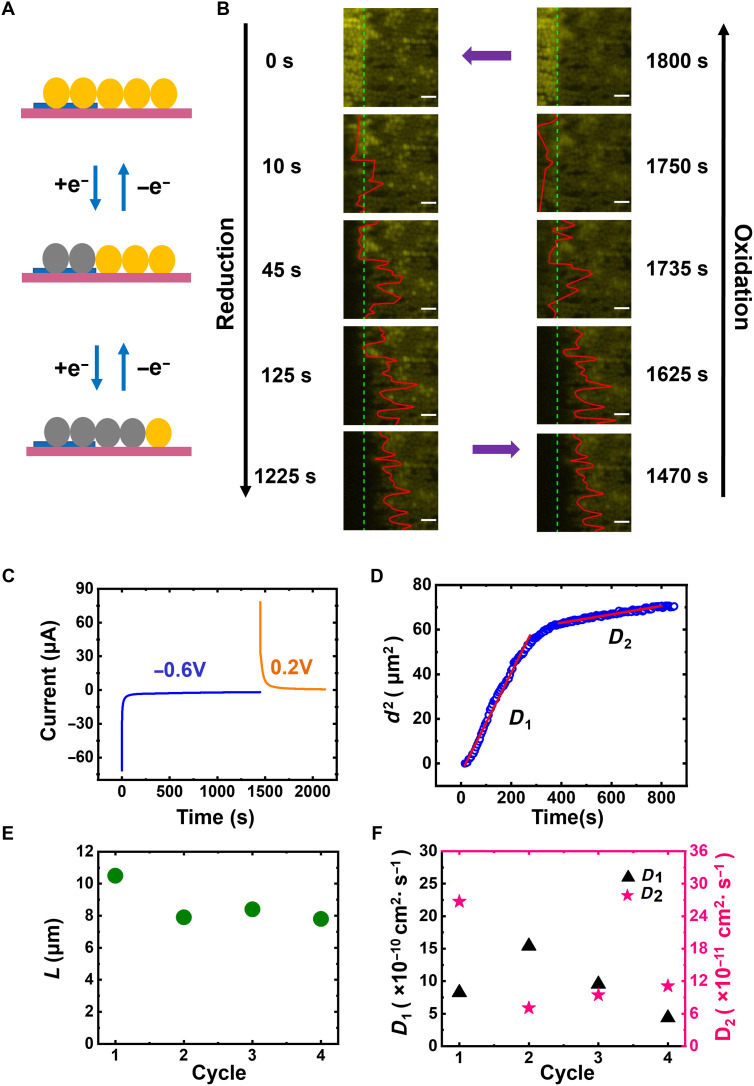
Intercolloid electron diffusion. (**A**) Schematic illustrating interparticle lateral charge transport (yellow: oxidized RAC; gray: reduced RAC; blue: metallic electrode; pink: glass slide). (**B**) Fluorescence images with fluorescence front tracking, red line, extracted from movie S2, during reduction and oxidation. Scale bars, 4 μm. Colloids above the platinum electrode appear brighter than those above the glass substrate because of reflection from the electrode and possibly metal-enhanced fluorescence. (**C**) Chronoamperometry (CA) performed on a RAC monolayer, with reduction potential held at −0.6 V versus Ag/AgCl and oxidation potential held at +0.2 V versus Ag/AgCl. (**D**) Tracking result from movie S2: lateral distance squared versus time (plotted from fig. S17), with least-squared linear fits for two sections. (**E**) Lateral quenched limit (*L*) tracked for four cycles on the same sample. (**F**) Lateral charge-transport diffusion coefficients (*D*_1_ and *D*_2_) determined as shown in (D); note the different *y*-axis scales.

In the previous section, RACs were in direct contact with an underlying electrode. Colloids in the near vicinity but not contacting the electrode also exhibit fluorescence switching, so long as the colloids form a percolating pathway to the electrode. Using this discovery, in situ fluorescence imaging is used to visualize interparticle electron transport during electrochemical cycling. Assuming electron transport occurs via thermally activated electron hopping as described by Dahms and Ruff (*35*, *36*), charge transport will occur between physically touching colloids (Fig. 3A). To quantify interparticle energy transport, the front edge of the fluorescence pattern is tracked (Materials and Methods and fig. S16) as the electrode potential is switched between reducing and oxidizing (Fig. 3C) values.

By synchronizing the movie with the electrochemical potential (movie S2), the relationship between the electrode potential and redox state of the colloids both in direct contact and connected via a percolating pathway with the electrode is determined. In Fig. 3B, the RACs are initially in the oxidized (fluorescent) state. At *t* = 0 s, the electrode is switched to −0.6 V. The fluorescence of RACs on the electrode are substantially quenched within ~10 s. By ~100 s, the fluorescence front propagates past the edge of the electrode (denoted by the green dashed line in Fig. 3B and Materials and Methods) into the percolating colloids on the glass. After about 800 s, the front stops moving (fig. S17). When the electrode potential is switched to +0.2 V, the fluorescence front retreats toward the substrate with RACs on the electrode recovering last (Fig. 3B). The fluorescence front propagates multiple colloid diameters away from the electrode, suggesting interparticle electron transport. To verify the requirement for RACs to be in physical contact for energy transfer to occur in control experiments where isolated clusters of RACs were present near, but not touching the electrode, the fluorescence of these clusters does not change during electrochemical cycling (fig. S18 and movie S3), providing strong evidence that energy transport is not occurring via a solvent-mediated process.

The reduction cycle is relatively easy to understand. RACs in direct contact with the electrode are reduced first, and then subsequently, RACs increasingly farther away from the electrode are reduced. The oxidation cycle is a bit more complex. Naively, one would assume that RACs directly in contact with the electrode should switch first and RACs away from the electrode last. However, that is not the case. The RACs furthest from the electrode switch first, and those in contact with the electrode last. It is important to remember that RACs only fluoresce when almost fully oxidized (Fig. 1G). We believe that the switched colloids furthest from the electrode are only slightly reduced, and thus when the electrode potential is switched, it is those RACs that fluoresce first and then the colloids above the electrode, which are the most fully reduced (oxidized colloids even further away from the electrode can also serve as a sink for electrons). During oxidation, we sometimes did observe RACs on the electrode recovering their fluorescence almost immediately. We assume that these are the particles that are not percolated into the RAC network and thus oxidize more rapidly than RACs around them.

We note that over multiple cycles, after ~800 s, the fluorescence front appears to stop 8 to 10 μm away from the edge of the electrode (Fig. 3E and fig. S17). The data in Fig. 3D correspond to cycle 3 (movie S2) in both Fig. 3E and 3F. We can only speculate why the front stops moving 8 to 10 μm from the electrode edge, but we think the most likely possibility is the impact of cumulative defects (e.g., gaps in the monolayer which eventually stops energy transfer)

$$d^{2}=2D_{\text{CT}}t$$ (3)

While we can only speculate why fluorescence quenching stops 8 to 10 μm from the electrode, it is still possible to quantify CT kinetics in the RAC monolayer. By plotting the lateral propagation distance squared (*d*^2^) against time (*t*) (Fig. 3D), we observe two distinct Fickian regions (*24*) (Eq. 3) with least-squared linear fits, with slopes of 0.229 μm^2^/s (up to ~270 s) and 0.0189 μm^2^/s (from about 400 to 800 s). We suspect the change in slope, and the eventual stop after about 800 s is due to a breakdown in percolation. We note that the front, red line in Fig. 3B, is jagged, as would be expected for a weakly percolated system (see fig. S17 for more details). Because the slope is twice the numerical value of the effective CT diffusion coefficient *D*_CT_, we calculate *D*_1_ to be 1.15 × 10^−9^ cm^2^/s and *D*_2_ to be 9.45 × 10^−11^ cm^2^/s. These values are within the same orders of magnitude as reported for other viologen-containing polymers (*10*, *20*, *37*) (10^−11^ to 10^−10^ cm^2^/s), giving us confidence that our physical interpretation of the data is reasonable. *D*_1_ and *D*_2_ over four cycles are shown in Fig. 3F.

### Energy transfer in a RAC suspension

**Fig. 4.**
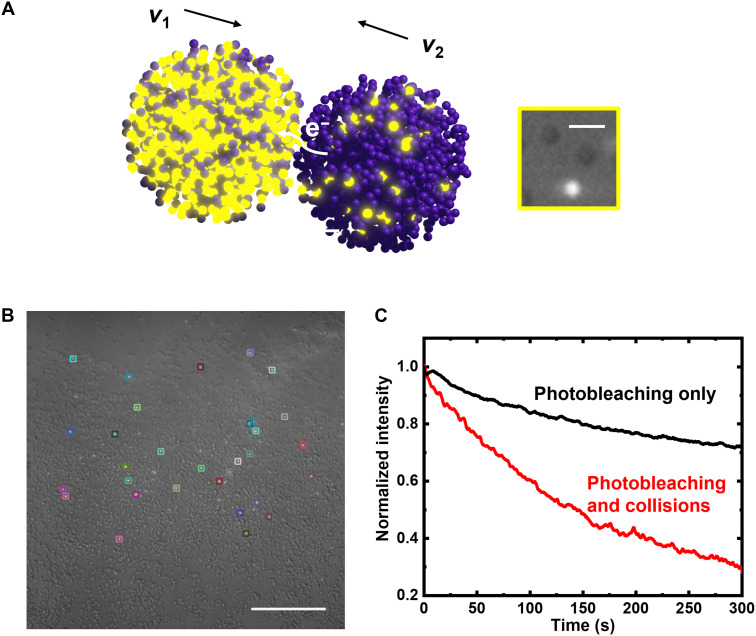
Collision-induced energy exchange. (**A**) Schematic of net electron exchange between two colliding RACs at different oxidation states (pendant groups not drawn to scale) and optical microscopy image of oxidized (bright) and reduced (dark) RAC when both transmitted light and fluorescence excitation are turned on for imaging. (**B**) Thirty-one discrete oxidized RAC (color-boxed) pinned to the substrate. (**C**) Fluorescence intensity (normalized) change (red) over 300 s of the 31 oxidized particles in (B) and six oxidized particles (separate experiment) that underwent photobleaching only (black). Scale bars, 3 μm (A) and 50 μm (B).

Imaging a suspension of RACs at different redox states enables indirect visualization of electron transport upon particle-particle collisions (Fig. 4A). A reduced RAC suspension with an effective concentration of 10 mM EV groups is sealed in an airtight microchamber with glass slide, plastic spacer (1.0 mm), and epoxy glue in an argon-filled glovebox. Via imaging both in transmission and fluorescence modes, oxidized and reduced particles are easily distinguished (Fig. 4A, inset). We find a region in the field of view where 31 discrete particles are oxidized due to slow redox kinetics with Zn powder (Fig. 4B, with oxidized RAC labeled by boxes) and adsorbed on the glass substrate. We track individually these oxidized RAC’s light intensity (Materials and Methods) as they are randomly contacted by freely diffusing reduced particles (movie S4). During imaging, the fluorescence of the pinned particles decreases by >71% within 300 s (red curve in Fig. 4C and Materials and Methods) due to both electron exchange between RACs and photobleaching. Control experiments conducted on six discrete oxidized particles that only undergo photobleaching show fluorescence decrease averaging only <29% (black curve in Fig. 4C, Materials and Methods, and movie S5) under equal or greater laser intensity, strong evidence for energy transfer between colliding RACs at varying oxidation states. In a more dilute suspension, energy transfer appears to be correlated with the number of collisions as oxidized RACs’ fluorescence intensity decreases as they appear to collide with reduced particles (fig. S19, movie S6, and Materials and Methods).

## DISCUSSION

Because redox-active polymers bear charges at highly localized sites and conduct electricity via electron transfer between these sites (*8*, *38*), in contrast to conducting polymers that conduct electricity down a conjugated backbone, redox-active polymers are both defect-tolerant and can be processed via typical methodologies. For example, RAC, formed of crosslinked nonconjugated redox-active polymer chains, can be synthesized via common solution-based methods. Similar electrically conducting colloids would be quite difficult to form from conjugated polymers. These RACs are unique in that they can exchange electrical energy upon contact with an electrically conductive substrate and each other and that their optical properties are highly dependent on their redox state, enabling an optical window into their charge-transport properties. Exploiting their highly nonlinear RAC electrofluorochromism, redox-based intercolloid energy transport in a RAC monolayer is directly observed, providing a simple method to determine *D*_CT_ from real-space imaging. We note that *D*_CT_ here is a convolution of both intracolloid and intercolloid electron hopping, and we do not know which of these two processes is rate-determining. While decoupling intra- and intercolloid resistance is challenging, advanced imaging techniques with higher spatial and temporal resolutions [e.g., super-resolution microscopy (*39*–*41*)] may in the future enable decoupling intra- and intercolloid electron transport.

By relating oxidation state with fluorescence intensity in a densely packed, percolated RAC monolayer, the quantification of the kinetics of electron transport through colloid-metal and colloid-colloid contacts is realized. The observed ~10-μm-lateral charge transport in nonconjugated polymers is an order of magnitude greater than previously observed in other radical polymers (*8*, *10*) and is probably made possible by the fact that solvent swells the RACs, greatly increasing the dynamics of the redox-active pendant groups. Energy transfer is also observed in a suspension of colliding RACs. As mentioned in Introduction, one future research area is to study the impact of doping (*4*–*6*, *13*) on RAC conductivity and CT length. We hope that this work serves as a starting point to understand electrochemical energy transfer in RACs, which may affect systems as diverse as redox flow batteries, electrochemical sensors (*42*), organic electronics (*43*), and colloidal robotics (*44*, *45*).

## MATERIALS AND METHODS

### Materials

Unless otherwise stated, all starting materials and reagents were purchased from Sigma-Aldrich and used as received without further purification. Super P (99%, Alfa Aesar), reference electrode silver paste (Ag/AgCl, 65:35; Creative Materials), photoresist (AZ5214E, MicroChemicals), acetone (99.5% or over; Fisher Chemical), and isopropanol (99.9%; Fisher Chemical) were purchased and used as received without further purification. Water used in this work was nanopure water (18.2 megohm∙cm at 25°C) purified by the Milli-Q Advantage A10 system. All air or moisture-sensitive manipulations were performed under nitrogen atmosphere using standard schlenk techniques. All glassware was oven-dried before use.

### Synthesis of RAC particles

RACs were synthesized (fig. S1) and characterized via scanning electron microscopy, dynamic light scattering, and infrared spectroscopy (fig. S2) following our previously published procedures (*10*).

#### Polyvinylbenzyl chloride particles (750 nm)

Polyvinylpyrrolidone (1.15 g, average molecular weight of 40 kDa) was added to 95 ml of 200-proof EtOH in a 200-ml Morton flask with a mechanical stirrer. The mixture was stirred at ~165 rpm and purged with nitrogen for 15 min before heating at 70°C. Then, AIBN (0.10 g) was dissolved in a mixture of 4-vinylbenzyl chloride (4.9 ml, 90%) and divinylbenzene (0.10 ml, 80%) and added into the reaction mixture. After 12 hours, the reaction mixture was centrifuged, and the supernatant was decanted. The particles were used directly in the next step without further purification or drying.

#### Redox-active colloid

Dry argon-flushed Dimethylformamide (DMF) (45 ml) and tetrahydrofuran (45 ml) were added to a flask containing polyvinylbenzyl chloride colloidal particles (1.5 g), and the resultant mixture was sonicated in bath sonicator to obtain a homogeneous dispersion (~ 1 hour). Then, ethyl viologen (15 g) was added to the dispersion. The resultant mixture was purged with nitrogen for about 20 min and then stirred at 90°C for 7 days. After cooling down to room temperature, saturated ammonium hexafluorophosphate aqueous solution was added to the above reaction mixture. To the resultant solution, DMF (20 ml) and acetonitrile (20 ml) were added and stirred at room temperature for 2 days. Crude product was obtained by adding water until the precipitate was observed. The crude product was separated by centrifuging. To the separated product, methanol was added and centrifuged, and the supernatant was decanted. This procedure was repeated three times with methanol and two times with ether, and the resultant product was dried under vacuum for 2 days. RACs are yellow-orange color.

### Photolithography procedures for device fabrication and electron-beam deposition

The three-electrode device for in situ imaging is fabricated in the cleanroom as follows. The electrodes are patterned onto glass slides with dimensions of 75 mm by 50 mm. The glass slides are cleaned with acetone, isopropanol (IPA), deionized (DI) water, and IPA in sequence with squeeze bottles and blown dry with industrial grade N_2_. The glass slide is then dehydrated on a hotplate at 110°C for 5 min. Photoresist (AZ5214E) is spin-coated onto the glass slide with spin speed of 3000 rpm for 30 s. The glass slides are then baked on a hotplate at 110°C for 1 min and then exposed under ultraviolet (UV) light for 9 s using a photomask and developed using AZ917 for 55 s. The glass slides are immediately immersed in a DI water bath for a few seconds and then blown dry with N_2_. After development, the inverse image of the electrode is already visible to the eye, and the devices are baked on a hotplate at 110°C for 3 min. After photolithography, 50-nm Pt is deposited using electron-beam evaporation on top of 5-nm Cr as adhesion layer. Subsequently, the device is sonicated in acetone for 5 min, leaving behind only the metallic electrodes (fig. S6B).

### Preparation of CE and pseudo-RE

To prepare the counter electrode (CE), carbon black (super P, 99%), and polyvinylidene fluoride are mixed with a few drops of *N*-methylpyrrolidone (NMP) (99%) using a pestle and mortar for 20 min. Then, a small piece of slurry is cast on one of the Pt electrodes, and the substrate is heated at 205°C for 45 min to ensure NMP removal. After letting the device cool down in ambient air for 10 min, Ag/AgCl (65:35) is deposited as a paste on the device as the RE and cured for 30 min at 175°C.

### Pseudo-RE potential calibration

On the fabricated device with the pasted pseudo-reference Ag/AgCl electrode (RE), CV experiments (20 mV/s) are run in a 10 mM ferrocene solution with 0.1 M LiBF_4_ as supporting electrolyte in acetonitrile. Based on redox peaks, ferrocene oxidation takes place at ~0.59 V versus RE (fig. S7), which according to literature (*46*) indicates the EV first reduction occurring at ~−0.22 V versus RE.

### Fluorescence imaging of the RAC

The fluorescence imaging experiments are carried out on a ZEISS LSM7 LIVE system with a 20× (EC Plan-Neofluar 20×/0.50 M27), 40× (Plan-Apochromat 40×/1.4 Oil DIC M27), or 100× (Plan-Apochromat 100×/1.40 Oil M27) objectives depending on different magnification requirements in different experiments. The wavelength of the excitation laser is 488 nm, and a filter is applied to block all the light with wavelengths below 495 nm. The glass slide is mounted on the sample holder and connected to the portable potentiostat with copper tape. Figure S6D shows a photo of the setup for in situ fluorescence imaging while conducting electrochemical cycling with a portable potentiaostat (BioLogic).

### Particle counting technique

Particle counting was conducted by a home-built MATLAB code suite. Different from the zoomed-in view shown in fig. S4, a large-scale scanning of the whole sample area was conducted using the “tiling” module in the confocal microscope to keep single-particle resolution (fig. S8). A Gaussian filter was first applied onto the original image (built-in function imgaussfilter.m with an SD value of 0.01). Then, centroid positions of each particle were tracked on the basis of the peak intensity positions of each particle using the codes developed for optical microscopy (*32*) [pkfnd.m and cntrd.m from (*32*)]. Tracking results and zoomed-in views of a typical region are shown in fig. S8. Red dots in fig. S8C denote centroid positions of single colloid, whose intensity values were tracked over time to extract the temporal intensity evolution in Fig. 1E and figs. S11A and S20B.

### Number of EV groups in a single RAC and on the entire electrode used to develop the working curves in Fig. 1G

Number of pendant groups in a single RAC (Dry state) = $\frac{\rho_{1}\times V\times N_{A}}{M_{w}}$ = 1.25 g/cm^3^ ×$\left[\times \frac{1}{6}\pi \times {\left(9.5\times 10^{-5}\text{cm}\right)}^{3}\right]\times$ 6.022×10^23^÷ 592 g/mol = 5.71 × 10^8^.

Total number of EV groups in 34731 particles = 5.71 × 10^8^ × 34731 ≈ 1.98 × 10^13^.

Dry state density of RAC was reported before (*10*).

### Tangent line fitting (*24*) for extracting Faradaic charges in the cyclic voltammetry plot

The Faradaic charges which are used for redox reactions as opposed to resistive or capacitive charges (non-Faradaic) are extracted from the CV plot displayed in the software EC-Lab (BioLogic). Demonstrated in fig. S9A, by subtracting the real-time trapezoidal area enclosed by *i*_1_, *i*_2_, *x* axis, and the tangent line from the integrated area of *I* versus potential (potential easily convertible to time if divided by scan rate), one can plot the *Q*_Faradaic_ over time as presented in fig. S11B. Quenching start point is chosen when the total fluorescence in the field of view decays no smaller than 5%, and quenching ending point is chosen when the total fluorescence in the field of view remains no larger than 6%. See fig. S9A (cycle 1 at 5 mV/s), where the quenching start potential is ~−0.19 V: We select 400-mV positive and 200-mV negative than this value of −0.19 V as the upper and lower bounds of linear fitting (61 data points in total, fig. S9B). For cycles 2 and 3, since the electrochemistry environment has shifted, we see a background shift (upward and leftward) in CV (fig. S5) compared with cycle 1. To ensure that the results of tangent line fitting make physical sense, the tangent point is moved 300 mV more negative (fig. S9, D and E). The number of data points selected (61) and the approach of choosing upper and lower bounds remain.

### Method for developing working curve of fluorescence intensity versus percentage of reduced functional groups in scattered RAC particles

In fig. S11A, green dashed lines denote the starting and ending of quenching in cycle 1 at a sweep rate of 5 mV/s. In this interval, normalized intensity versus time data with reduced viologen groups (%) versus time data is superimposed, so that a double *y*-axes plot is obtained (fig. S11B). In addition, with correlating the two *y* axes, a working curve emerges (fig. S11C).

### Calculating chemical quenching ability of free-flowing reduced EV molecules (EV^+^·) in a RAC dispersion containing 10 mM oxidized viologen groups

After an initial addition of 8.19 × 10^−5^ M reduced EVs to the RAC dispersion (10 mM EV groups), PL emission intensity around 570 nm decreases (fig. S14) from 1799 (arbitrary units) to 632 (arbitrary units): one can calculate that one EV^+^·molecule approximately quenches 10 mM ×(1799-632) ÷ 1799 ÷(8.19 × 10^−5^ M) ≈ 79 pendant groups (EV^2+^).

### PL measurement and time-resolved PL measurement procedures

The PL spectra are taken at room temperature, using a NKT super continuum laser (SuperK Extreme), filtered to 488 nm with a bandwidth of 5 nm and total power average on the sample of 2 mW. The laser is focused on the sample by a parabolic mirror, which is also used to collect the luminescence from the sample. The collected luminescence is directed through a long -pass filter with a cut-off wavelength of 530 nm, to eliminate the scattered laser light from the sample. For spectral measurements, the PL light is directed to an astigmatism-corrected Princeton Instruments (Spectra Pro 500i) 500-mm focal length spectrometer, equipped with a charge-coupled device camera. The time-correlated single-photon counting measurements are performed using a Si single-photon counting avalanche photodiode and a Becker & Hickl TCSPC module (SPC-130).

### Bulk electrolysis (reduction) of RACs and UV-Vis measurements of reduced RAC

Electrolysis was performed on a CHI760 potentiostat and in an O_2_ and moisture free environment inside of an Ar-filled drybox. A three-compartment cell was used, with the WE in the center compartment, the two lateral compartments occupied by a carbon felt as a counter electrode, and a nonaqueous Ag/Ag^+^ reference electrode (0.1 M AgNO_3_, MeCN). A carbon felt on Pt wire was used as the WE and held at a constant overpotential while stirring. Current and charge response over time are recorded. For reduction, the potential is held −150 mV from *E*_1/2_. UME (ultramicroelectrode) voltammograms using 12.5-μm-radius Pt UMEs are obtained before and after electrolysis to track steady-state limiting currents, confirming change of oxidation state (fig. S21). UV-visible (UV-Vis) absorption spectra (blue curve in Fig. 2B) are recorded on an HP8452A Diode-Array Spectrophotometer using a 1-cm pathlength quartz cuvette. Dispersions of reduced RAC are diluted to 0.1 mM in acetonitrile, and spectra are collected.

### Propagation front tracking of RAC monolayer scenario

Fluorescence front in a colloidal monolayer was tracked by a home-built MATLAB code suite. Shown in fig. S16, the electrode boundary position was first tracked by turning on the transmission so that electrode will block the light and show as dark. The fluorescence front inside the colloidal monolayer was tracked by the intensity difference between colloids that are reduced (dark) and oxidized (bright). Details of the tracking algorithm are given below. The electrode boundary was first tracked from the optical images by scanning over the intensity line-by-line. From the optical image captured before reduction happens, a Gaussian filter (built-in MATALB function: imgaussfilt.m) with an SD of 5 was applied to smooth image. For each horizontal line along the *x* axis (blue line), the fluorescence intensity profile is averaged by neighboring five pixels (red dots). The averaged intensity was then fitted by a tangent-hyperbolic (*47*) function: $f\left(x\right)=A+B\times \text{tanh}\left[\left(x-x_{0}\right)/w\right]$ , in which A , B , $x_{0}$ , and w were four independent fitting parameters and $x_{0}$ was determined as the electrode boundary position for one fixed y coordinate (blue diamond). By applying this method to each *y* coordinate, the electrode boundary profile was extracted showing as red line, and the approximate boundary was calculated from the linear regression of the boundary profile (green dotted line). Fluorescence wavefront beyond electrode in RAC monolayer was tracked following a similar algorithm used to map the electrode profile stated above. Starting from the original image, a Gaussian filter with an SD of 3 was applied, and the positions of the fluorescence front are extracted from this image by scanning the intensity profile of each horizontal line. From the intensity profile of a line scan, the intensity was fitted by the same tangent-hyperbolic function: $f\left(x\right)=A+B\times \text{tanh}\left[\left(x-x_{0}\right)/w\right]$ , and $x_{0}+w$ was determined as the position of fluorescence front for one-fixed *y* coordinate (blue diamond). It is noteworthy that here, the fluorescence front was selected at the very edge when the fluorescence intensity begins to decrease. By applying this method to each *y* coordinate, the fluorescence front profile was extracted showing as red line.

### Fluorescence intensity tracking of RACs with both photobleaching and collisions

With a laser of 40% maximum power continuously turned on throughout the 300-s experiment, we measured the intensity decrease of those pinned oxidized RACs (movie S4), which were likely caused by both photobleaching effect and collisions. To track the intensity change of those pinned RACs, centroid coordinates of 31 RACs were first manually annotated using ImageJ. Then, the mean intensity within a 2 × 2 pixel area adjacent to each centroid coordinate was recorded throughout the movie to obtain 31 intensity–time curves. Each curve was normalized by dividing the mean intensity within the initial 10 s of each curve. Last, all 31 curves were averaged to give the normalized intensity change curve with both photobleaching and collision, shown as the red curve in Fig. 4C.

### Fluorescence intensity tracking of RACs undergoing photobleaching only

The normalized fluorescence intensity change of RACs with only photobleaching effect was obtained from two other videos containing static RACs without any collision, where 40 or 50% (movie S5) maximum laser power, which is equal to or greater than the data with collisions, was used. In total, six RACs were tracked, normalized, and averaged following the same method as above, shown as the black curve in Fig. 4C.

### Fluorescence intensity tracking of RACs with intermittent laser power

To correlate the RAC intensity change with collision with minimal photobleaching effect, the laser was occasionally turned on (20% maximum laser power) for around 4 s every 120 s during the total experimental time of 600 s (so the laser was turned on six times at 0, 120, 240, 360, 480, and 600 s). The scattering plot in fig. S19B showing the relationship between intensity change and number of collisions was collected from nine moving or static oxidized RACs in three experiments (movie S6 shows one of the experiments). First, the centroid coordinates of the RACs were manually measured every second throughout the movies using ImageJ, and meanwhile, the number of collisions of each RAC within the second was also manually counted. The fluorescence intensity values of the RACs were obtained by searching for the maximum pixel intensity within a 5 × 5 pixel area adjacent to the centroid coordinate of each particle at 0, 120, 240, 360, 480, and 600 s when the laser was turned on. The intensity decrease of each RAC between six time points with the laser turned on was calculated and normalized by the initial intensity value of the particle, resulting in five data points per particle. The normalized intensity change was then plotted against the summed number of collisions of the RAC within the corresponding time interval to obtain fig. S19B. By binning the collisions (bin size: 30), the decrease in fluorescence intensity versus number of collisions becomes more apparent (fig. S19C). Note that no error bar on the data point in fig. S19C indicates only one data point in the respective bin.

## References

[1] M. Berggren, G. G. Malliaras, How conducting polymer electrodes operate. Science 364, 233–234 (2019). 31000650 10.1126/science.aaw9295

[2] L. Luo, S. H. Choi, C. D. Frisbie, Probing hopping conduction in conjugated molecular wires connected to metal electrodes. Chem. Mater. 23, 631–645 (2011).

[3] M. E. Lyons, *Electroactive Polymer Electrochemistry: Part 1: Fundamentals* (Springer, 2013).

[4] A. G. Baradwaj, S. H. Wong, J. S. Laster, A. J. Wingate, M. E. Hay, B. W. Boudouris, Impact of the addition of redox-active salts on the charge transport ability of radical polymer thin films. Macromolecules 49, 4784–4791 (2016).

[5] E. P. Tomlinson, M. E. Hay, B. W. Boudouris, Radical polymers and their application to organic electronic devices. Macromolecules 47, 6145–6158 (2014).

[6] M. E. Hay, S. Hui Wong, S. Mukherjee, B. W. Boudouris, Controlling open-shell loading in norbornene-based radical polymers modulates the solid-state charge transport exponentially. J. Polym. Sci. B 55, 1516–1525 (2017).

[7] S. Mukherjee, *Organic Radical Polymers: New Avenues in Organic Electronics* (Springer, 2017).

[8] Y. Joo, V. Agarkar, S. H. Sung, B. M. Savoie, B. W. Boudouris, A nonconjugated radical polymer glass with high electrical conductivity. Science 359, 1391–1395 (2018). 29567710 10.1126/science.aao7287

[9] X.-H. Jin, M. B. Price, J. R. Finnegan, C. E. Boott, J. M. Richter, A. Rao, S. M. Menke, R. H. Friend, G. R. Whittell, I. Manners, Long-range exciton transport in conjugated polymer nanofibers prepared by seeded growth. Science 360, 897–900 (2018). 29798881 10.1126/science.aar8104

[10] E. C. Montoto, G. Nagarjuna, J. Hui, M. Burgess, N. M. Sekerak, K. Hernández-Burgos, T.-S. Wei, M. Kneer, J. Grolman, K. J. Cheng, J. A. Lewis, J. S. Moore, J. Rodríguez-López, Redox active colloids as discrete energy storage carriers. J. Am. Chem. Soc. 138, 13230–13237 (2016). 27629363 10.1021/jacs.6b06365

[11] G. Nagarjuna, J. Hui, K. J. Cheng, T. Lichtenstein, M. Shen, J. S. Moore, J. Rodríguez-López, Impact of redox-active polymer molecular weight on the electrochemical properties and transport across porous separators in nonaqueous solvents. J. Am. Chem. Soc. 136, 16309–16316 (2014). 25325703 10.1021/ja508482e

[12] I. E. Jacobs, A. J. Moulé, Controlling molecular doping in organic semiconductors. Adv. Mater. 29, 1703063 (2017). 10.1002/adma.201703063 28921668

[13] M. Ishii, Y. Yamashita, S. Watanabe, K. Ariga, J. Takeya, Doping of molecular semiconductors through proton-coupled electron transfer. Nature 622, 285–291 (2023). 37821588 10.1038/s41586-023-06504-8

[14] W. B. Russel, D. A. Saville, W. R. Schowalter, Colloidal dispersions, in *Cambridge Monographs on Mechanics and Applied Mathematics* (Cambridge Univ. Press, 1989).

[15] Q. Chen, S. C. Bae, S. Granick, Directed self-assembly of a colloidal kagome lattice. Nature 469, 381–384 (2011). 21248847 10.1038/nature09713

[16] Q. Chen, J. K. Whitmer, S. Jiang, S. C. Bae, E. Luijten, S. Granick, Supracolloidal reaction kinetics of janus spheres. Science 331, 199–202 (2011). 21233384 10.1126/science.1197451

[17] M. Burgess, J. S. Moore, J. Rodríguez-López, Redox active polymers as soluble nanomaterials for energy storage. Acc. Chem. Res. 49, 2649–2657 (2016). 27673336 10.1021/acs.accounts.6b00341

[18] H. Dahms, Electronic conduction in aqueous solution. J. Phys. Chem. 72, 362–364 (1968).

[19] I. Ruff, V. J. Friedrich, K. Demeter, K. Csillag, Transfer diffusion. II., Kinetics of electron exchange reaction between ferrocene and ferricinium ion in alcohols. J. Phys. Chem. 75, 3303–3309 (1971).

[20] Z. T. Gossage, N. B. Schorr, K. Hernández-Burgos, J. Hui, B. H. Simpson, E. C. Montoto, J. Rodríguez-López, Interrogating charge storage on redox active colloids via combined raman spectroscopy and scanning electrochemical microscopy. Langmuir 33, 9455–9463 (2017). 28621544 10.1021/acs.langmuir.7b01121

[21] H. Al-Kutubi, H. R. Zafarani, L. Rassaei, K. Mathwig, Electrofluorochromic systems: Molecules and materials exhibiting redox-switchable fluorescence. Eur. Polym. J. 83, 478–498 (2016).

[22] B. Doppagne, M. C. Chong, H. Bulou, A. Boeglin, F. Scheurer, G. Schull, Electrofluorochromism at the single-molecule level. Science 361, 251–255 (2018). 30026221 10.1126/science.aat1603

[23] M. Burgess, E. Chénard, K. Hernández-Burgos, G. Nagarjuna, R. S. Assary, J. Hui, J. S. Moore, J. Rodríguez-López, Impact of backbone tether length and structure on the electrochemical performance of viologen redox active polymers. Chem. Mater. 28, 7362–7374 (2016).

[24] A. J. Bard, *Electrochemical Methods: Fundamentals and Applications*. L. R. Faulkner, Ed., (Wiley, 1980).

[25] Y. Cao, A. Aspuru-Guzik, Accelerating discovery in organic redox flow batteries. Nat. Comput. Sci. 4, 89–91 (2024). 38388845 10.1038/s43588-024-00600-z

[26] T. Janoschka, N. Martin, U. Martin, C. Friebe, S. Morgenstern, H. Hiller, M. D. Hager, U. S. Schubert, An aqueous, polymer-based redox-flow battery using non-corrosive, safe, and low-cost materials. Nature 527, 78–81 (2015). 26503039 10.1038/nature15746

[27] H. Park, G. Kwon, H. Lee, K. Lee, S. Y. Park, J. E. Kwon, K. Kang, S. J. Kim, In operando visualization of redox flow battery in membrane-free microfluidic platform. Proc. Natl. Acad. Sci. U.S.A. 119, e2114947119 (2022). 35197286 10.1073/pnas.2114947119 PMC8892322

[28] A. A. Wong, S. M. Rubinstein, M. J. Aziz, Direct visualization of electrochemical reactions and heterogeneous transport within porous electrodes in operando by fluorescence microscopy. Cell Rep. Phys. Sci. 2, 100388 (2021).

[29] F. Miomandre, P. Audebert, Eds. in *Luminescence in Electrochemistry: Applications in Analytical Chemistry, Physics and Biology* (Springer, 2017).

[30] P. Audebert, F. Miomandre, Electrofluorochromism: From molecular systems to set-up and display. Chem. Sci. 4, 575–584 (2013).

[31] C. Lei, D. Hu, E. J. Ackerman, Single-molecule fluorescence spectroelectrochemistry of cresyl violet. Chem. Commun. , 5490–5492 (2008). 10.1039/b812161c 18997928

[32] J. C. Crocker, D. G. Grier, Methods of digital video microscopy for colloidal studies. J. Colloid Interface Sci. 179, 298–310 (1996).

[33] J. Keizer, Nonlinear fluorescence quenching and the origin of positive curvature in Stern-Volmer plots. J. Am. Chem. Soc. 105, 1494–1498 (1983).

[34] J. R. Lakowicz, *Principles of Fluorescence Spectroscopy* (Springer, ed. 3, 2006).

[35] D. N. Blauch, J. M. Saveant, Dynamics of electron hopping in assemblies of redox centers. Percolation and diffusion. J. Am. Chem. Soc. 114, 3323–3332 (1992).

[36] T. W. Kemper, R. E. Larsen, T. Gennett, Relationship between molecular structure and electron transfer in a polymeric nitroxyl-radical energy storage material. J. Phys. Chem. C 118, 17213–17220 (2014).

[37] E. F. Dalton, R. W. Murray, Viologen(2+/1+) and viologen(1+/0) electron-self-exchange reactions in a redox polymer. J. Phys. Chem. 95, 6383–6389 (1991).

[38] J. Lutkenhaus, A radical advance for conducting polymers. Science 359, 1334–1335 (2018). 29567695 10.1126/science.aat1298

[39] O. Nevskyi, D. Wöll, 3D super-resolution fluorescence imaging of microgels. Annu. Rev. Phys. Chem. 74, 391–414 (2023). 36750411 10.1146/annurev-physchem-062422-022601

[40] H. Park, D. T. Hoang, K. Paeng, L. J. Kaufman, Localizing exciton recombination sites in conformationally distinct single conjugated polymers by super-resolution fluorescence imaging. ACS Nano 9, 3151–3158 (2015). 25743935 10.1021/acsnano.5b00086

[41] D. Wöll, C. Flors, Super-resolution fluorescence imaging for materials science. Small Methods 1, 1700191 (2017).

[42] T. G. Drummond, M. G. Hill, J. K. Barton, Electrochemical DNA sensors. Nat. Biotechnol. 21, 1192–1199 (2003). 14520405 10.1038/nbt873

[43] S. R. Forrest, M. E. Thompson, Introduction: Organic electronics and optoelectronics. Chem. Rev. 107, 923–925 (2007).

[44] A. T. Liu, M. Hempel, J. F. Yang, A. M. Brooks, A. Pervan, V. B. Koman, G. Zhang, D. Kozawa, S. Yang, D. I. Goldman, M. Z. Miskin, A. W. Richa, D. Randall, T. D. Murphey, T. Palacios, M. S. Strano, Colloidal robotics. Nat. Mater. 22, 1453–1462 (2023). 37620646 10.1038/s41563-023-01589-y

[45] Z. Xu, T. Hueckel, W. T. M. Irvine, S. Sacanna, Transmembrane transport in inorganic colloidal cell-mimics. Nature 597, 220–224 (2021). 34497391 10.1038/s41586-021-03774-y

[46] R. R. Gagne, C. A. Koval, G. C. Lisensky, Ferrocene as an internal standard for electrochemical measurements. Inorg. Chem. 19, 2854–2855 (1980).

[47] T. O. E. Skinner, D. G. A. L. Aarts, R. P. A. Dullens, Grain-boundary fluctuations in two-dimensional colloidal crystals. Phys. Rev. Lett. 105, 168301 (2010). 21231020 10.1103/PhysRevLett.105.168301

