# Supplementary material for: Visualizing energy transfer between redox-active colloids
Source: Sci Adv. 2025 Sep 3;11(36):eady7716. doi: 10.1126/sciadv.ady7716 (PMC12407080; doi:10.1126/sciadv.ady7716)
Supplement: Supplementary file 1 — Figs. S1 to S21 Legend for movies S1 to S6 [file sciadv.ady7716_sm.pdf]

Science Advances
AAAS

Supplementary Materials for

Visualizing energy transfer between redox-active colloids

Alan Subing Qu *et al.*

Corresponding author: Qian Chen, qchen20@illinois.edu; Paul V. Braun, pbraun@illinois.edu

Sci. Adv. **11**, eady7716 (2025)
DOI: 10.1126/sciadv.ady7716

The PDF file includes:

Figs. S1 to S21
Legend for movies S1 to S6

Other Supplementary Material for this manuscript includes the following:

Movies S1 to S6

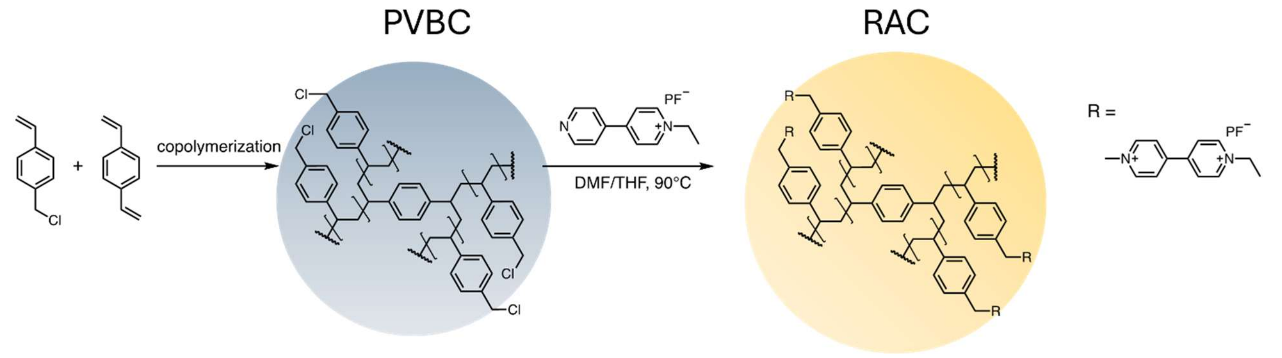

Fig. S1. Synthesis route of RACs.

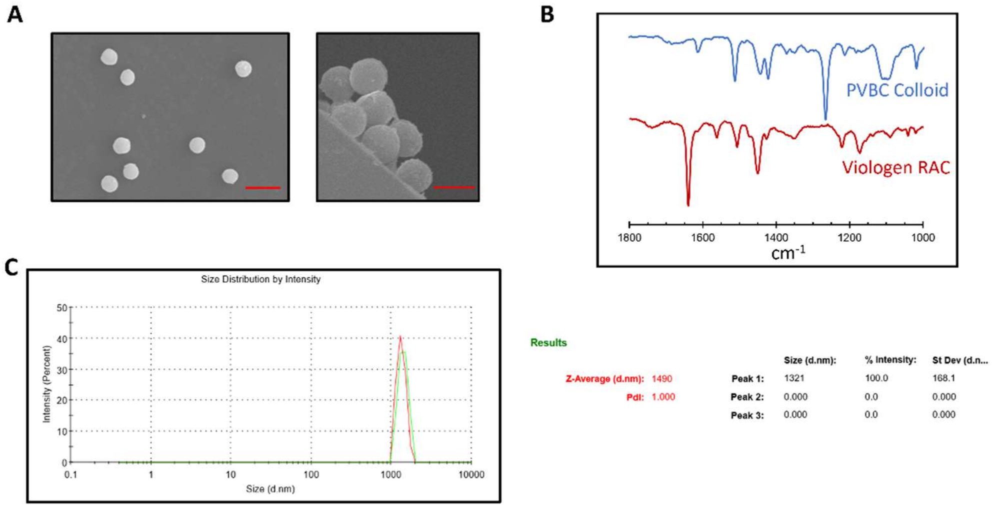

Fig. S2. Sizing and functional group characterizations of RACs. (A) Dry state Scanning Electron Microscopy (SEM). Averaged over 50 particles, the diameter is 950±32 nm. Scale bars: 2 µm (left), 1 µm (right). **(B)** Attenuated total reflection infrared spectra (ATR-FTIR) of pristine crosslinked poly (vinylbenzyl chloride) (xPVBC) colloids and ethyl-viologen derivatized colloids (RACs), showing that C-Cl bond stretching(*10*) (1240 cm^{-1}) is dampened after viologen substitution as well as the appearance of C-cationic N^+ vibrational modes(*10*) (1640 cm^{-1}). **(C)** Dynamic light scattering (DLS) measurements of 0.1 g/L oxidized RAC size in acetonitrile. Green: raw data; Red: fitting of raw data into a single peak at 1321±168 nm.

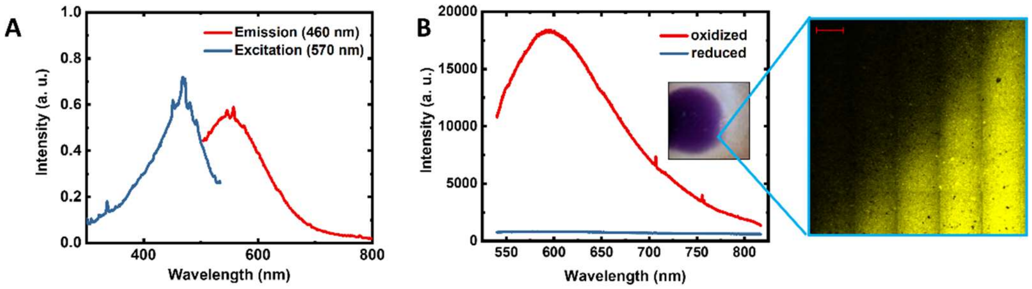

Fig. S3. Photophysical properties of RACs and electrochromism of its acetonitrile suspension (partly reduced by Zn). (A) Emission and excitation scan data obtained from a fluorometer on an oxidized RAC suspension (10 mM viologen groups). **(B)** Photoluminescence (PL) emission of oxidized and reduced RACs. Inset shows electrochromism of the RAC suspension, with violet region indicating reduced RACs (by zinc powder) and brighter orange-ish region indicating oxidized RACs. On the right is a zoomed-in fluorescence image. Scale bar: 20 µm. Note, stripes are artifacts due to inhomogeneity of the optical field.

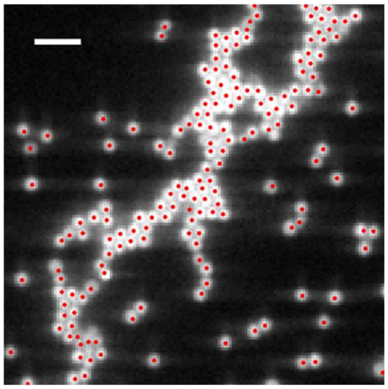

Fig. S4. Particle-counting corresponding to data in Fig. 1E. 166 particles in total, scale bar: 5 μm.

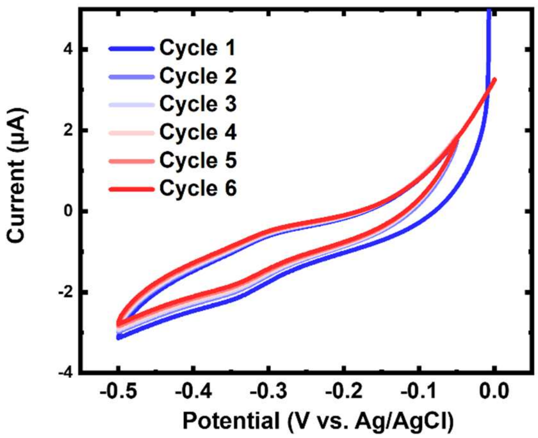

Fig. S5. Six-round cyclic voltammetry of RACs on the working electrode (total number: 34,731) at sweep rate 5 mV/s.

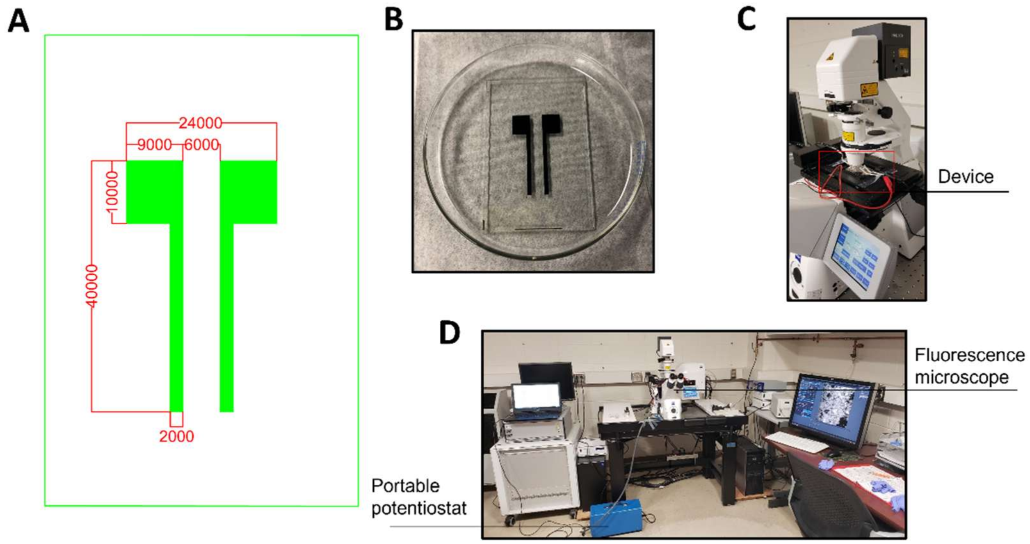

Fig. S6. Device design, device picture and experimental setup. (A) Photomask design drawn in CAD. Dimensions in μm. **(B)** Fabricated Pt electrodes on the glass slide. (**C)** Device mounted on the imaging stage of a fluorescence microscope. **(D)** Experimental set-up including a fluorescence microscope, a portable potentiostat, the device and a laptop.

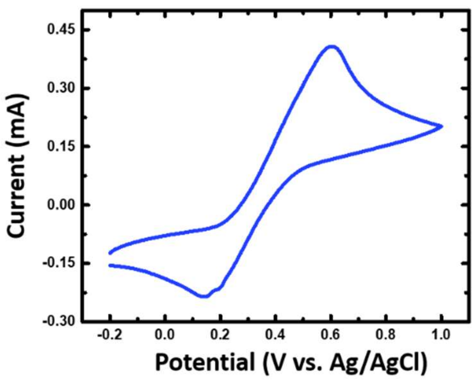

Fig. S7. Ferrocene cyclic voltammetry (20 mV/s) in the device used for fluorescence imaging.

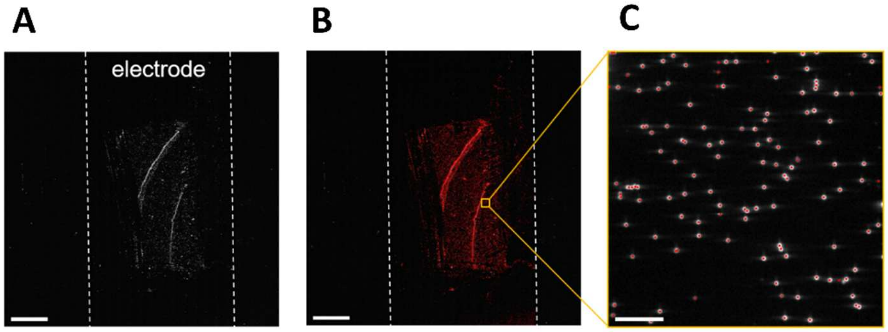

Fig. S8. Tracking and counting the total number of RACs on the electrode. (A) Raw fluorescence image of colloids on the electrode. The doted lines denote the boundary of the electrode. **(B)** Raw fluorescence image overlaid with particles on electrode tracked shown as the red dots (34,731 colloids). **(C)** Zoomed-in view showing a typical region on the electrode with centers of the colloids highlighted by red dots. Scale bars: 500 µm in **(A)**, **(B)** and 20 µm in **(C)**.

A 5 mV/s_Cycle 1 (with fluorescence quenching onset and completion noted)

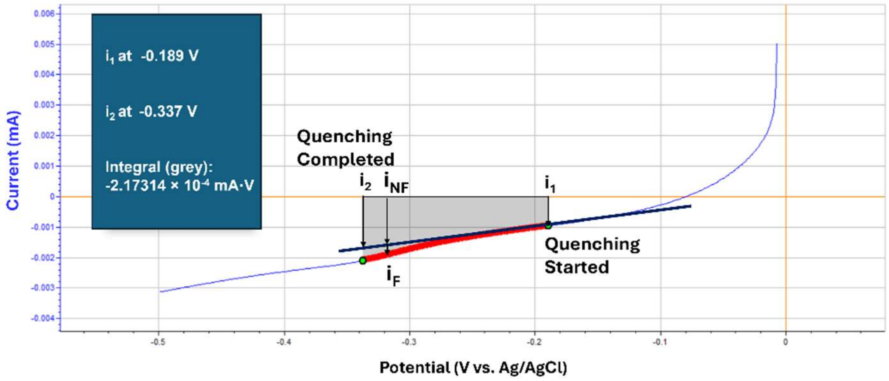

B 5 mV/s_Cycle 1 linear fitting

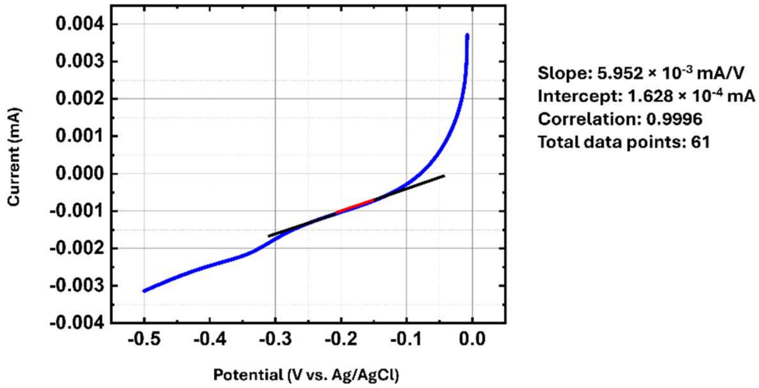

C 10 mV/s_Cycle 1 linear fitting

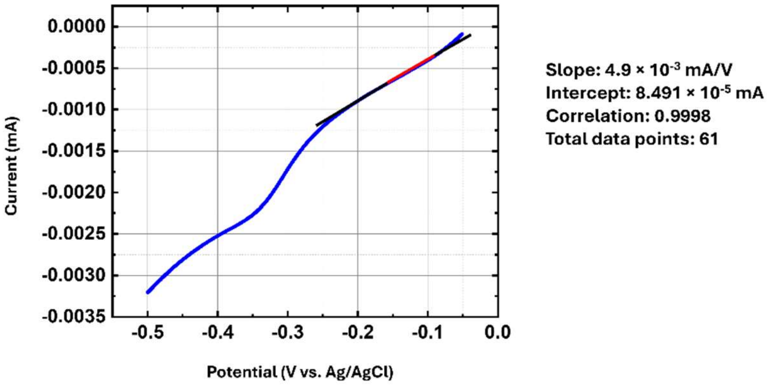

D **5 mV/s_Cycle 2 linear fitting**

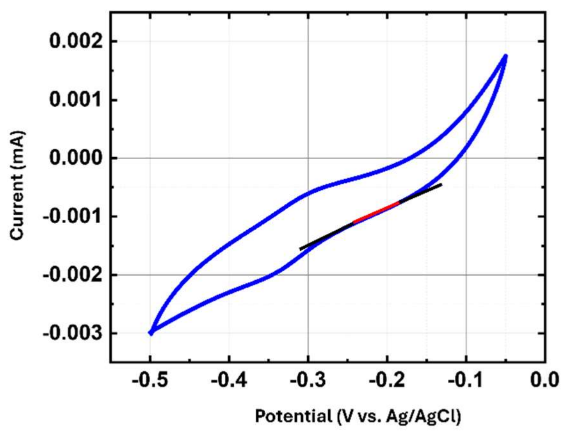

Slope: 6.201 × 10^{-3} mA/V
Intercept: 3.78 × 10^{-4} mA
Correlation: 0.9998
Total data points: 61

E **5 mV/s_Cycle 3 linear fitting**

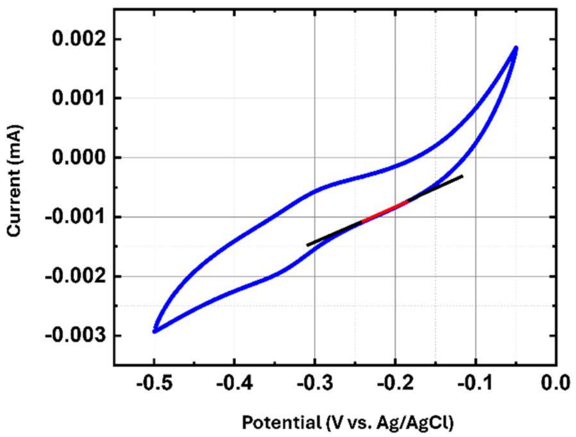

Slope: 6.206 × 10^{-3} mA/V
Intercept: 4.037 × 10^{-4} mA
Correlation: 0.9997
Total data points: 61

Fig. S9. Tangent line drawings and schematics of how to extract Faradaic charges from cyclic voltammetry plots. (A) Demonstration of raw data processing for Cycle 1 at 5 mV/s as an example, where i_1 and i_2 represent the quenching onset and finishing point currents. i_{NF} denotes non-Faradaic current, and i_F denotes the Faradaic counterpart. Software interface: EC-Lab®, BioLogic. **(B) – (E)** Linear fittings for CV data in 4 scenarios with results on the right. Each fitting uses 61 data points (red) for method internal consistency.

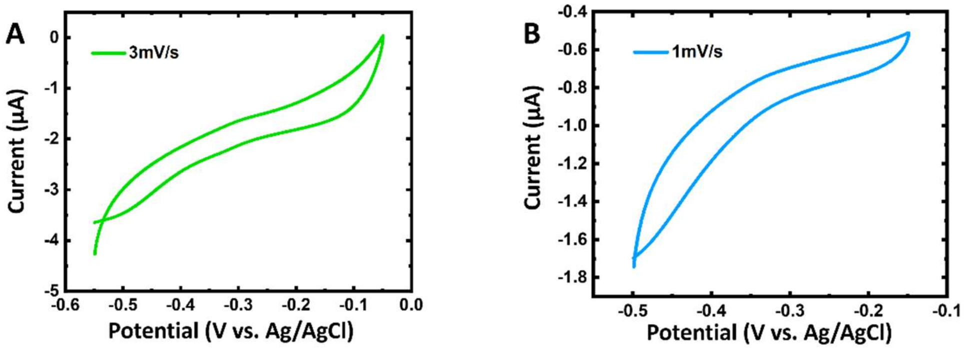

Fig. S10. Lower sweep rates cyclic voltammetry of RACs on the working electrode. (A) sweep rate: 3 mV/s. **(B)** sweep rate: 1 mV/s. RAC casting concentration and method are the same as shown in Fig. S8.

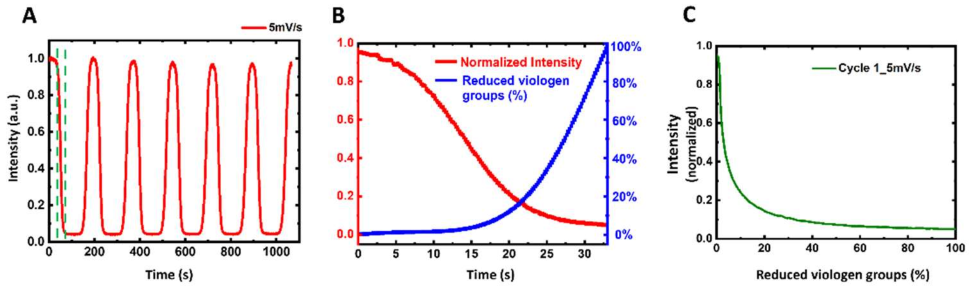

Fig. S11. Process of developing working curve for RACs from Cycle 1 at 5 mV/s. All the other working curves presented in Fig. 1G and Fig. S13 are developed using the same workflow.

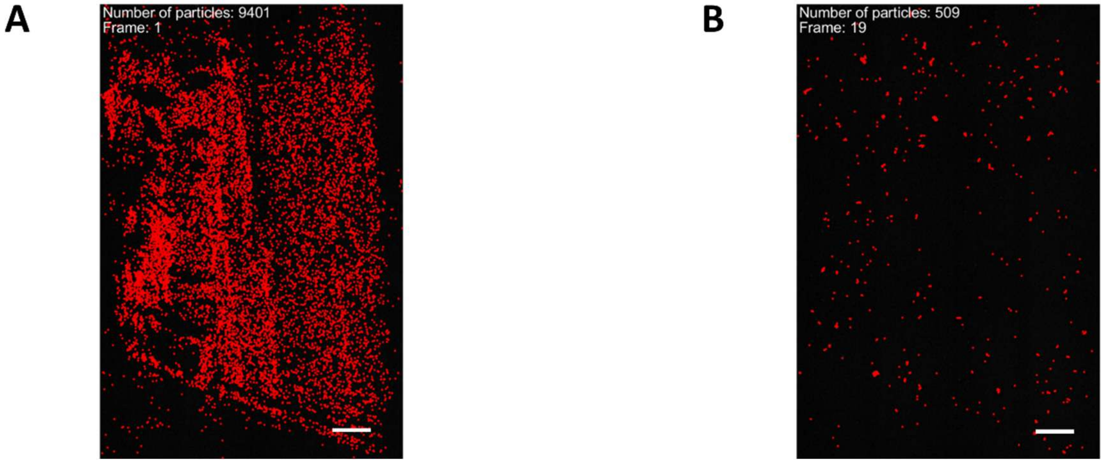

Fig. S12. Low-magnification (5X) large-area imaging before (A) and after (B) reducing RACs on the entire working electrode (sweep rate: 10 mV/s). (A) Particle counting result: 9401. **(B)** Particle counting result: 509. Scale bars: 200 µm.

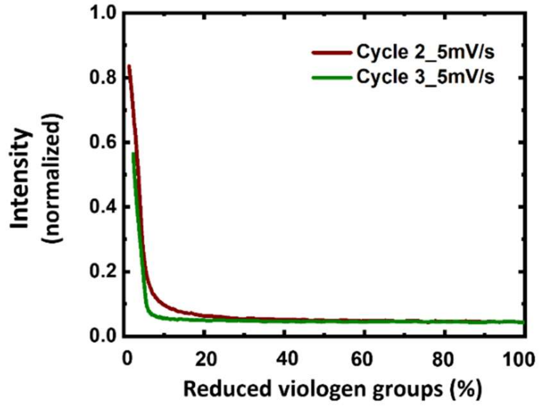

Fig. S13. Fluorescence intensity (normalized) vs. percentages of reduced pendant groups in RACs extracted from Cycles 2 and 3 at a 5 mV/s sweep rate.

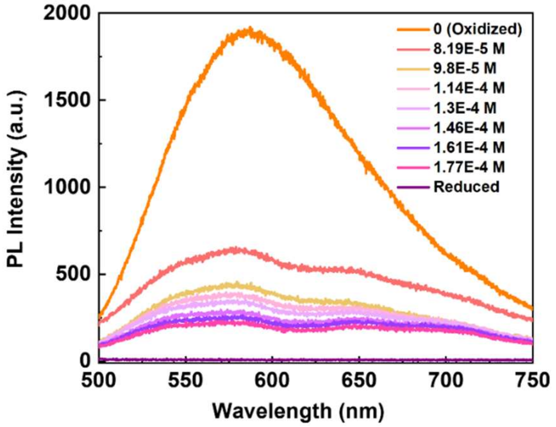

Fig. S14. Photoluminescence emission data of oxidized RAC dispersion (10 mM viologen groups) with stepwise additions of reduced ethyl-viologen small molecules ($EV^{+\cdot}$).

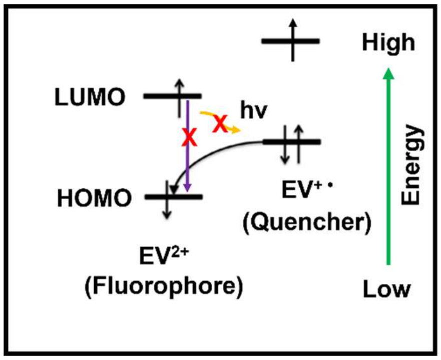

Fig. S15. Proposed electron state diagram for electron-transfer based quenching in RAC system. When an EV^{2+} is excited, one of the paired electrons in $EV^{+\bullet}$ can transfer to the now available highest occupied molecular orbital (HOMO) of the excited EV^{2+}, inhibiting relaxation of the excited electron from the EV^{2+} lowest unoccupied molecular orbital (LUMO), quenching the fluorophore.

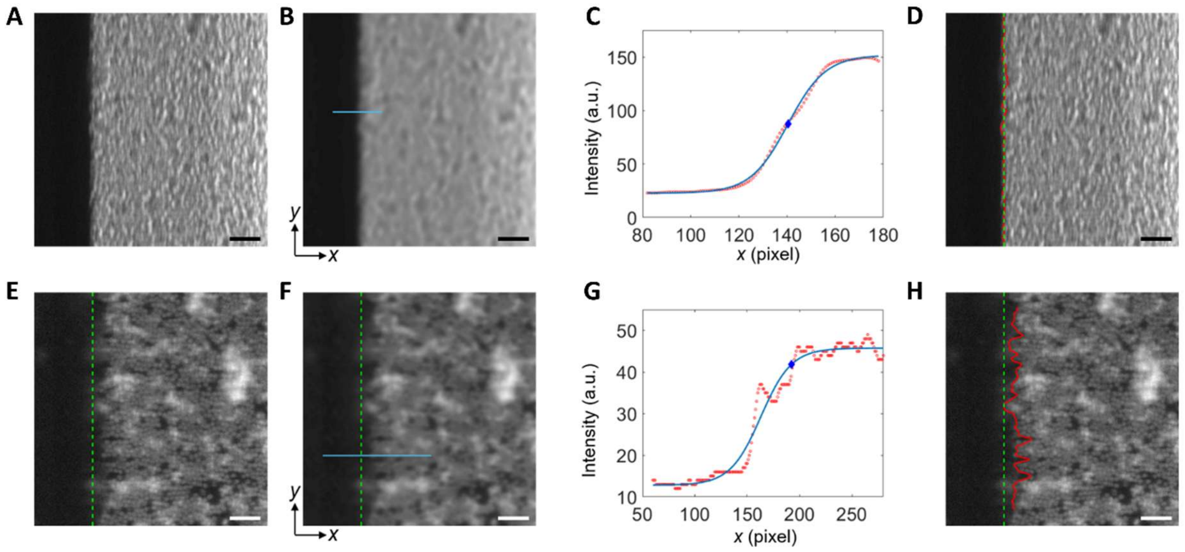

Fig. S16. Automatic boundary detection of the electrode periphery and fluorescence pattern front for RAC monolayer. (A) An optical image of RAC monolayer before reduction with transmitted light turned on. The black region is the electrode. **(B)** The filtered image of **(A)** for boundary detection. The blue line indicates one position of horizontal intensity scan. **(C)** Intensity profile along the blue line in **(B)** after smoothing over neighboring 5 pixels (red dots). Blue line is fitted line and the blue diamond indicates the position of the electrode periphery. **(D)** The original image overlaid with the exact (red solid) and approximate (green dashed) boundaries. **(E)** A fluorescence image of RAC film after 24.6 minutes of reduction. The green dotted line is the approximate boundary from **(D)**. **(F)** The filtered image of **(E)** for boundary detection. The blue line indicates one position of horizontal intensity scan. **(G)** Intensity profile along the blue line in **(F)** (red dots). Blue line is the fitted line and the blue diamond indicates the position of the fluorescence front. **(H)** The original image overlaid with the fluorescence front identified. Scale bars: 10 µm and single pixel size is 0.16 µm.

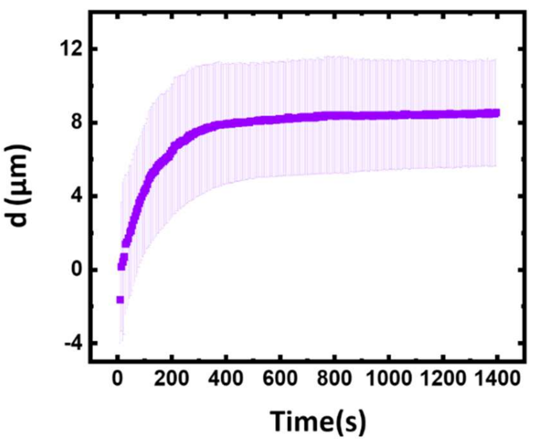

Fig. S17. Lateral distance (d) between fluorescence pattern front and Pt/glass boundary over time, for the reduction part in Fig 3C. Shaded area (with vertical bars) denotes the error bars, attributed to tracking multiple horizontal lines orthogonal to the fluorescence pattern front.

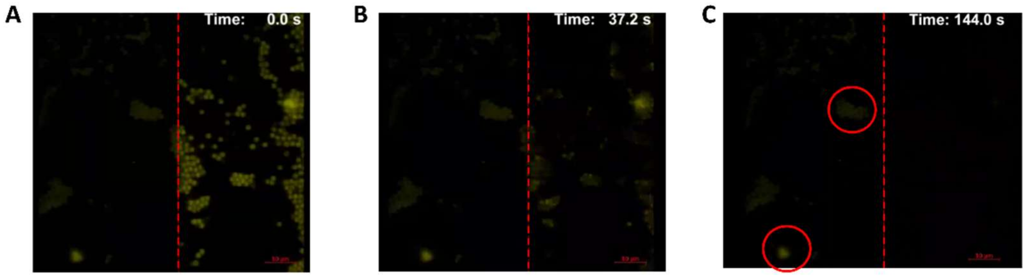

Fig. S18. Demonstration of no charge transport between non-percolating RAC clusters (sweep rate: 20 mV/s). Time-lapsed snapshots **(A) - (C)** of fluorescence quenching in a non-percolated RAC monolayer (Movie S3) extending beyond the electrode boundary. Note, the right side of the images is the electrode, and the left side is the insulating gap (glass slide as substrates), with red dashed line denoting electrode boundary. From **(C)** it is seen that isolated RAC clusters do not lose fluorescence emission after RAC on the electrode are fully reduced, indicating that electron transport requires physical contact between RACs.

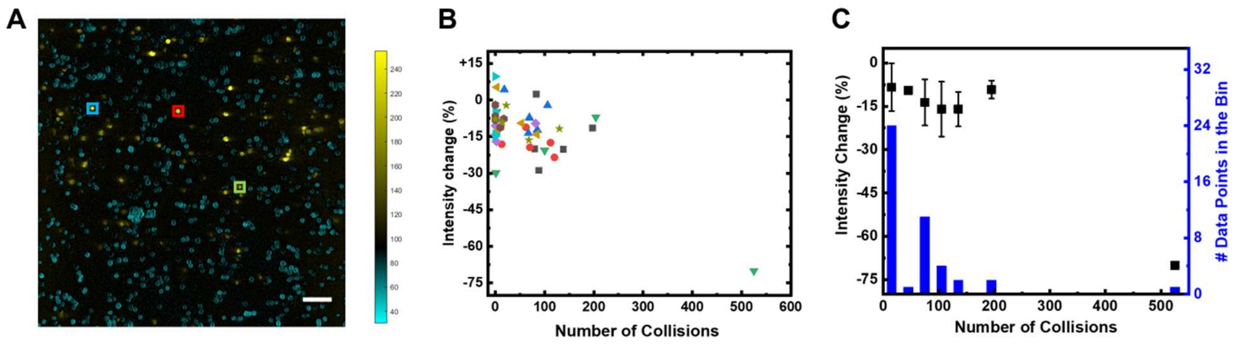

Fig. S19. Energy Transfer in a RAC suspension with number of collisions. (A) Dual transmission light and fluorescence imaging of 3 oxidized particles while diffusing among more reduced RACs (Movie S6). Scale bar: 10 µm. **(B)** Oxidized RACs' fluorescence intensity change vs. number of collisions with more reduced RACs. Data sampled from 9 initially oxidized particles, all tracked over 600 s. **(C)** Binning results of **(B)** with error bars, bin size: 30 collisions.

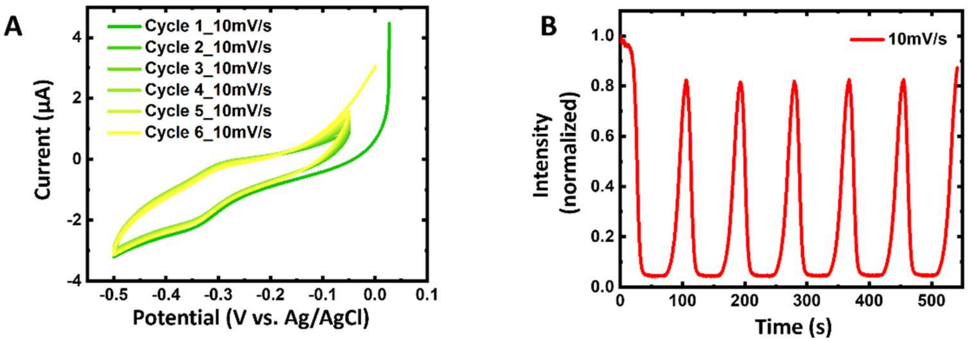

Fig. S20. Electrochemical cycling and optical imaging data used to develop the working curve at 10mV/s in Fig. 1G. (A) Six-round cyclic voltammetry run on 34,731 colloids. **(B)** Ensemble fluorescence intensity vs. time of the 166 particles in the field of view (Fig. S4). After the first round, fluorescence intensity does not return to the original level because this scan rate does not provide sufficient time to fully oxidize the RACs.

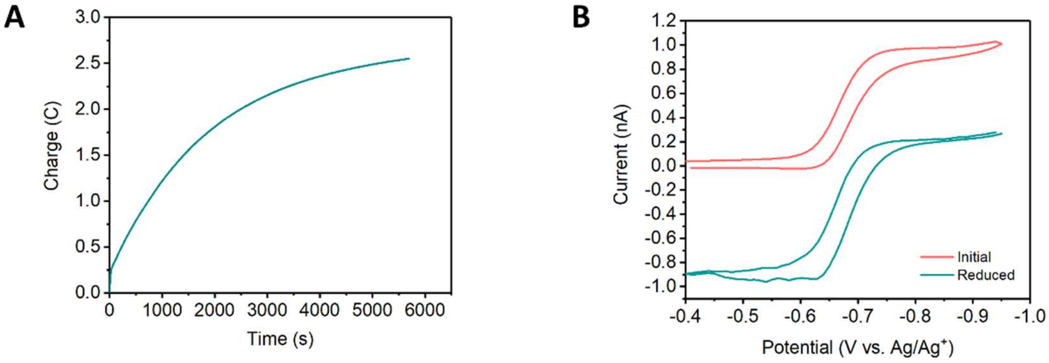

Fig. S21. Bulk electrolysis of RACs. (A) Charge vs. time relationship for electrolyzing 6.3 mM RACs (viologen groups) in 0.1 M $LiBF_4$ MeCN charged at -0.85 V vs. Ag/Ag^+. **(B)** UME-CVs (UME: ultramicroelectrode) show full conversion of RACs to the reduced state.

Movie S1.

Synchronized electrochemical cycling and optical imaging of RAC monolayer fluorescence switching on the working electrode at 1 mV/s sweep rate.

Movie S2.

Synchronized electrochemical and optical imaging of RAC monolayer lateral energy transport (Cycle 3 in Figs 3E, 3F).

Scale bar: 20 μm.

Movie S3.

Demonstration of no charge transport between non-percolating RAC clusters on the insulating glass substrate (imaged with cyclic voltammetry at 20 mV/s sweep rate).

Pt electrode is located on the right side of the field of view, whereas insulating glass is on the left side. Non-percolating RAC clusters on the glass do not lose fluorescence when particles on Pt are reduced.

Movie S4.

Light intensity tracking of 31 discrete oxidized RACs collided by surrounding reduced RACs, imaged with 40% maximum laser power.

Movie S5.

Fluorescence intensity tracking of 3 oxidized RACs which only underwent photobleaching, imaged with 50% maximum laser power.

Note, RACs on the left side of the field of view lose their fluorescence because they are seated on top of a platinum electrode and are electrochemically reduced during imaging.

Movie S6.

Light intensity tracking of 3 oxidized RACs in a more dilute suspension, imaged with 20% maximum laser power.

The intensity tracking is conducted while number of collisions is manually counted (Fig. S19). RACs are imaged with transmitted light (brightfield) always on and fluorescence emission excited six times ($t \approx 0$ s, 120 s, 240 s, 360 s, 480 s, 600 s) for approximately 4 s per interval. Gray baseline tracks average intensity in the field of view, indicating no apparent defocus during imaging.
